# ATG16L1 promotes cell migration and invasion in high glucose–induced retinal capillary endothelial cells

**DOI:** 10.3389/fmed.2025.1515936

**Published:** 2025-07-15

**Authors:** Xinxiao Gao, Pinxue Xie, Wen Feng, Jindong Han, Xiaobo Tan

**Affiliations:** ^1^Department of Ophthalmology, Beijing Anzhen Hospital, Capital Medical University, Beijing, China; ^2^Beijing Institute of Heart, Lung and Blood Vessel Diseases, Beijing Anzhen Hospital, Capital Medical University, Beijing, China; ^3^Department of Ophthalmology, Xiangya Hospital, Central South University, Changsha, China; ^4^Tianjin Key Laboratory of Retinal Functions and Diseases, Tianjin Branch of National Clinical Research Center for Ocular Disease, Eye Institute and School of Optometry, Tianjin Medical University Eye Hospital, Tianjin, China; ^5^Department of Ophthalmology, The Affiliated Hospital of Chengde Medical University, Chengde, China

**Keywords:** ATG16L1, diabetic retinopathy, cell migration, cell invasion, retinal capillary endothelial cells

## Abstract

**Purpose:**

Diabetic retinopathy (DR), a common chronic complication of diabetes, often results in irreversible visual dysfunction. This study investigated the mechanisms underlying the expression of ATG16L1, a potential biomarker of DR.

**Methods:**

We investigated the role of ATG16L1 in high glucose (HG)–induced retinal capillary endothelial cells (RCECs). Rat RCECs were cultured in normal glucose (NG) or HG medium with or without ATG16L1 transfection.

**Results:**

The mRNA and protein expression levels of ATG16L1 were significantly upregulated in RCECs exposed to HG medium. The migration ability and invasion rate of RCECs were significantly higher in the HG group than in the NG group but decreased markedly after transfection with ATG16L1 siRNA, compared with those in the control group (*p* < 0.01).

**Conclusion:**

ATG16L1 might be involved in the development of DR by promoting the migration of RCECs.

## Introduction

Diabetic retinopathy (DR) is a chronic complication of diabetes that often results in irreversible loss of vision. The prevalence of DR in patients with diabetes for ≥15 years is as high as 98%, making it the most common cause of blindness in the working population of developed countries (1, 2). DR gradually progresses from normal to nonproliferative DR and eventually to proliferative DR (PDR). In the PDR stage, blood and extracellular fluid leak out due to the instability of retinal neovascularization, resulting in vitreous hemorrhage and retinal detachment, which can eventually cause blindness (3). Consequently, researchers have extensively studied the pathogenesis of DR to determine effective methods for preventing, treating, and reducing visual dysfunction.

The primary characteristics of DR include pericyte loss, basement membrane thickening, microaneurysm formation, neovascularization, and blood–retinal barrier disruption. The pathogenesis of DR is extremely complex, involving several different cells, multiple molecules, and various factors (4). Currently, the most studied mechanisms include an increase in the flux of the polyol or hexosamine pathway, an increase in the formation of advanced glycation end products, abnormal activation of signaling cascades, upregulation of oxidative stress, and peripheral nerve damage (5). However, owing to its complexity, the pathophysiological mechanisms of DR are not yet fully understood. Therefore, it is necessary to investigate the pathogenesis of DR to determine effective intervention methods and therapeutic targets.

To better understand the role of autophagy in DR, we previously evaluated the related genes via microarray analysis (6). The results of our Signalnet analysis showed that Bcl2, Gabarapl2, Atg4c, and ATG16L1 were involved in autophagy in DR. As the expression of ATG16L1 increased considerably, it might be an important marker for the development of DR. Therefore, to further elucidate the expression and role of ATG16L1 in DR, we investigated the effect of ATG16L1 transfection on the biological behavior of retinal capillary endothelial cells (RCECs).

## Materials and methods

### Cell culture and treatment

Rat RCECs were purchased from Saibaikang (Shanghai, China) and cultured in endothelial cell medium (ScienCell, United States) containing 5.5 mM glucose, 20% fetal bovine serum (FBS; Gibco, United States), 200 μg/mL heparin sodium, 400 μg/mL endothelial cell growth supplement (ScienCell, United States), 100 units/mL penicillin sodium, and 100 μg/mL streptomycin sulfate. Then, the cells were inoculated in a 0.5% gelatin flask at 37°C in a 5% CO_2_ incubator. After being cultured in six-well plates until a confluence of approximately 70%, the cells were transfected with chemically synthesized ATG16L1 siRNA, whereas the siRNA negative controls (siRNA NC) were transfected with Lipofectamine 3,000 (Thermo Fisher, United States) according to the manufacturer’s instructions. After 24 h of transfection, the cells were cultured separately in 5.5 mM (normal glucose [NG]) or 25 mM (high glucose [HG]) glucose medium for 24 and 72 h, respectively. Then, the cells were divided into the following six groups: (i) NG (*n* = 5); (ii) HG (*n* = 5); (iii) NG + siRNA NC (*n* = 5); (iv) HG + siRNA NC (*n* = 5); (v) NG + ATG16L1 siRNA (*n* = 5); and (vi) HG + ATG16L1 siRNA (*n* = 5).

### Real-time polymerase chain reaction

Total RNA was extracted from the cells using TRIzol reagent (Invitrogen, Carlsbad, Canada), and the obtained mRNA was reverse-transcribed using a reverse transcription kit (Takara, Shiga, Japan). Quantitative reverse transcription (qRT)–PCR assays were conducted on an ABI Prism 7500 sequence detection system (Applied Biosystems, Foster City, CA, United States) and repeated for each sample. The reaction mixture (20 μL) comprised 2 μL of cDNA template, 0.6 μL each of forward and reverse primers, and 10 μL of 2 × SYBR Green PCR Mix (Takara). Primers targeting ATG16L1 (F: 5′-GCAAGCCGAATCTGGACT-3′, R: 5′-CCTGAGACTATCCGTGCAT-3′) were used for real-time RT–PCR amplification. The specificity of PCR was determined via fusion curve analysis and gel electrophoresis, and data were analyzed using the standard curve method. *β*-actin (F: 5′-CCAGCCTTCCTTCTTGGGTA-3′, R: 5′-CAATGCCTGGGTACATGGTG-3′) was used as an internal reference, and the target gene level was calculated using the 2^−ΔΔCt^ method.

### Western blotting

After washing the cells thrice with phosphate-buffered saline (PBS), total protein was extracted using RIPA Biotechnology buffer (Beyotime Biotechnology, Shanghai, China). Protein concentration was measured using a bicinchoninic acid protein assay kit (Beyotime Biotechnology) according to the manufacturer’s protocol. Proteins were separated via 10% sodium dodecyl sulfate–polyacrylamide gel electrophoresis and transferred onto a polyvinylidene fluoride membrane (Millipore, Germany). The membrane was blocked with 5% skim milk at 37°C for 2 h and then incubated overnight at 4°C with the specific primary antibody ATG16L1 (Affinity, DF3825) and ACTIN (MDL, MDL11027). After washing the membrane thrice with 1 × Tris-buffered saline with Tween 20, it was incubated at 37°C for 2 h with a secondary antibody conjugated to horseradish peroxidase. The protein bands were visualized using enhanced chemiluminescence reagents.

### Wound healing assay

The cells were seeded into six-well plates and cultured until a confluence of approximately 90%. The cell layer was scratched using the tip of a sterile pipette to form a wound. Cellular debris was removed using PBS, and the medium was replaced. The cells were divided into different treatment groups and then incubated (5-well cells were grouped as one group, and there were a total of six groups). Images of the same field were acquired under a microscope (Carl Zeiss) at 0 and 48 h. Each experiment was repeated thrice. The percentage of wound healing was evaluated using ImageJ software (National Institutes of Health, United States).

### Transwell invasion assay

The cells in the different treatment groups were diluted to 1 × 10^5^/mL (5-well cells were grouped as one group, and there were a total of six groups). Then, 200 μL of the cell suspension in serum-free Dulbecco’s modified Eagle medium (DMEM) was added to the upper Transwell chamber coated with Matrigel, and 600 μL of DMEM containing 20% FBS was added to the lower chamber. After incubation at 37°C for 24 h, the invading cells were fixed with 4% paraformaldehyde and stained with 0.1% crystal violet. Each experiment was repeated thrice. Digital photographs were acquired, and the proportion of migratory cells was measured using ImageJ software (National Institutes of Health).

### Statistical analysis

Measurement data were presented as mean ± standard deviation. Independent Student’s *t*-tests were performed to compare normally distributed continuous variables between two groups. GraphPad Prism 7 (GraphPad Software, United States) was used for all statistical analyses and graph plotting. Chi-squared test or Fisher’s exact test was used to compare noncontinuous variables. The experiments were repeated at least three times independently. All statistical analyses were conducted using SPSS 20.0 (SPSS Inc., Chicago, IL, United States). Differences among and between groups were considered statistically significant at *p* < 0.05.

## Results

### Dynamic expression of ATG16L1 in RCECs cultured in HG medium and changes in the expression after siRNA transfection

**Figure 1:**
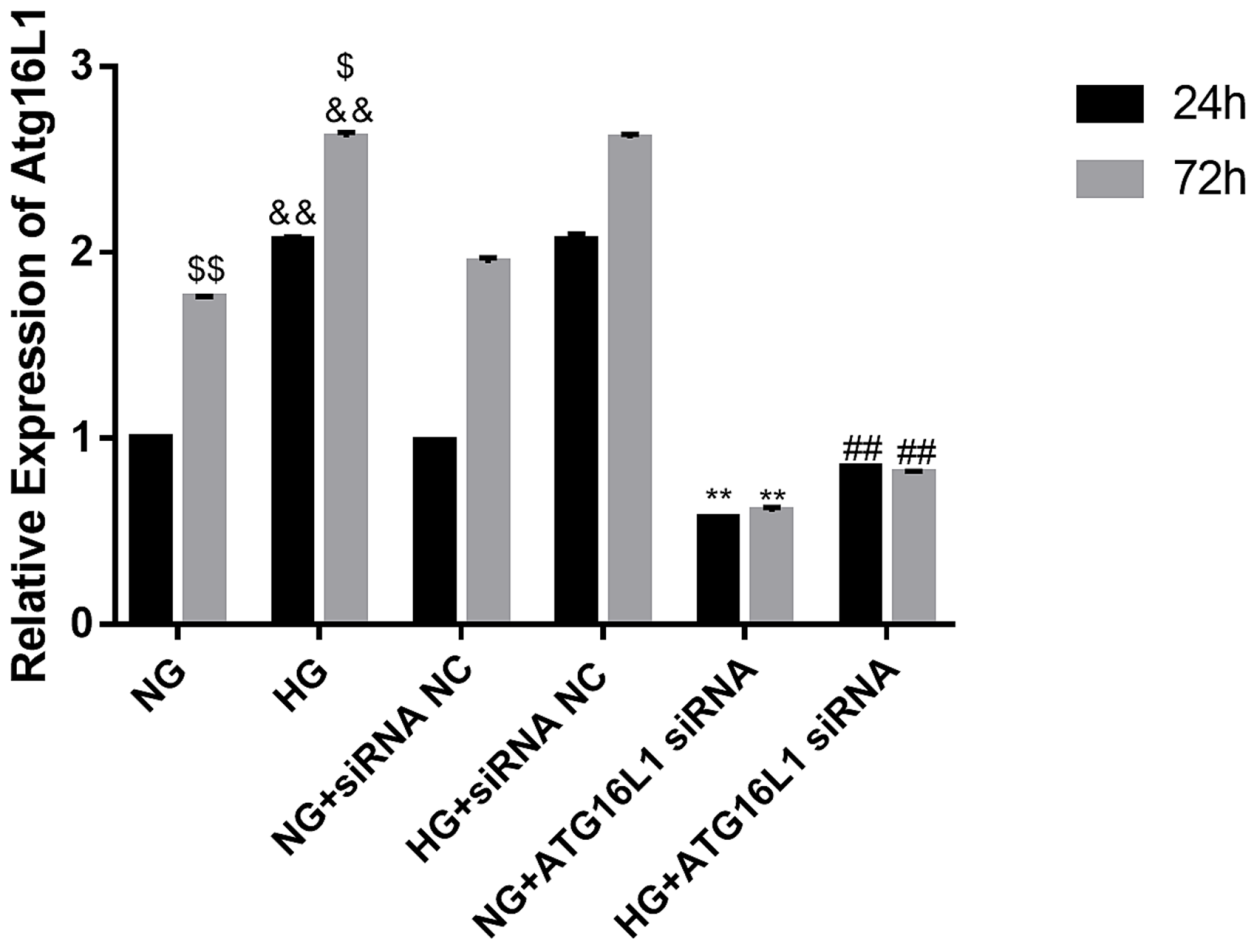
The expression of ATG16L1 increased after high glucose (HG) treatment. The expression of ATG16L1 siRNA decreased at both 24 and 72 h after treatment. &&: *p* < 0.01, compared with the normal glucose (NG) group. $$: *p* < 0.01, compared with the NG group at 24 h. $: *p* < 0.05, compared with the HG group at 24 h. **: *p* < 0.01, compared with the NG + siRNA negative control (NC) group. ##: *p* < 0.01, compared with the HG + siRNA NC group.

**Figure 2:**
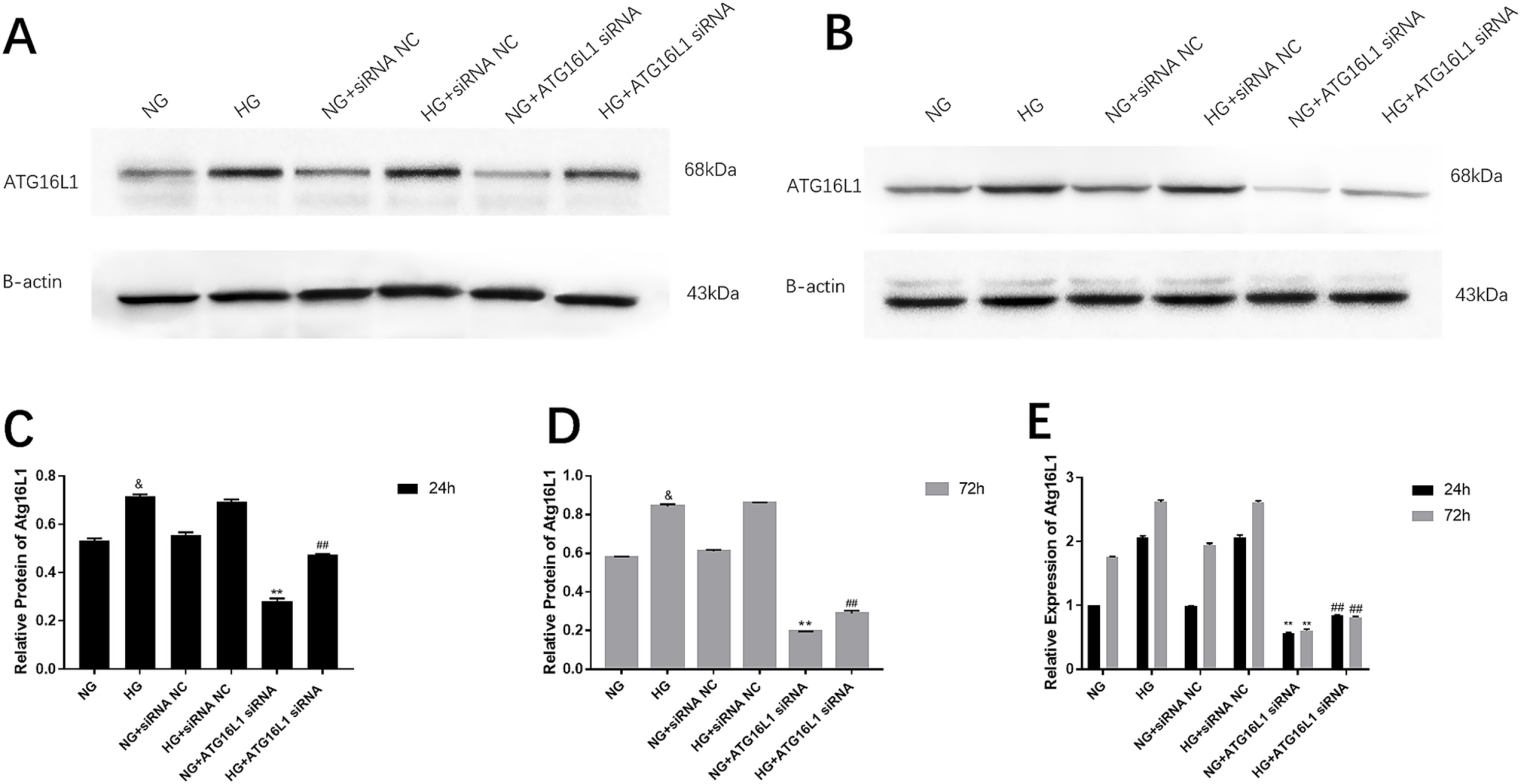
ATG16L1 protein expression in retinal capillary endothelial cells under high glucose (HG) conditions and changes in expression after siRNA transfection. **(A)** ATG16L1 protein expression in cells after culture for 24 h. **(B)** ATG16L1 protein expression in cells after culture for 72 h. **(C)** At 24 h of HG treatment, the protein expression of ATG16L1 increased. The protein expression decreased after treatment with ATG16L1 siRNA. &: *p* < 0.05, compared with the normal glucose (NG) group at 24 h. **: *p* < 0.01, compared with the NG + siRNA negative control (NC) group at 24 h. ##: *p* < 0.01, compared with the HG + siRNA NC group at 24 h. **(D)** At 72 h of HG treatment, the protein expression of ATG16L1 increased. The protein expression decreased after treatment with ATG16L1 siRNA. **(E)** Compared with treatment at 24 h, the protein expression of ATG16L1 significantly increased in the NG, HG, and NG + siATG16L1 groups at 72 h. &: *p* < 0.05, compared with the NG group at 72 h. **: *p* < 0.01, compared with the NG + siRNA NC group at 72 h. ##: *p* < 0.01, compared with the HG + siRNA NC group at 72 h.

After 24 and 72 h of culture, the mRNA expression of ATG16L1 in RCECs in the HG group was significantly upregulated compared with that in the NG group at the same time point, whereas it was significantly downregulated in the ATG16L1 siRNA group (0.987 ± 0.007, *n* = 5) (Figure 1). Furthermore, the mRNA expression of ATG16L1 in RCECs after 24 h exposure to both NG and HG was lower than that after 72 h exposure. The protein expression of ATG16L1 was upregulated in RCECs after HG treatment for 24 h (2.063 ± 0.021, *n* = 5) and 72 h (2.618 ± 0.027, *n* = 5) compared with NG treatment, whereas it was downregulated in the ATG16L1 siRNA group (Figures 2A–E). The protein expression of ATG16L1 in RCECs in the NG and HG groups was similar between the two treatment durations (24 vs. 72 h) (Figures 2C–E).

### Effect of ATG16L1 on the migration ability of RCECs

**Figure 3:**
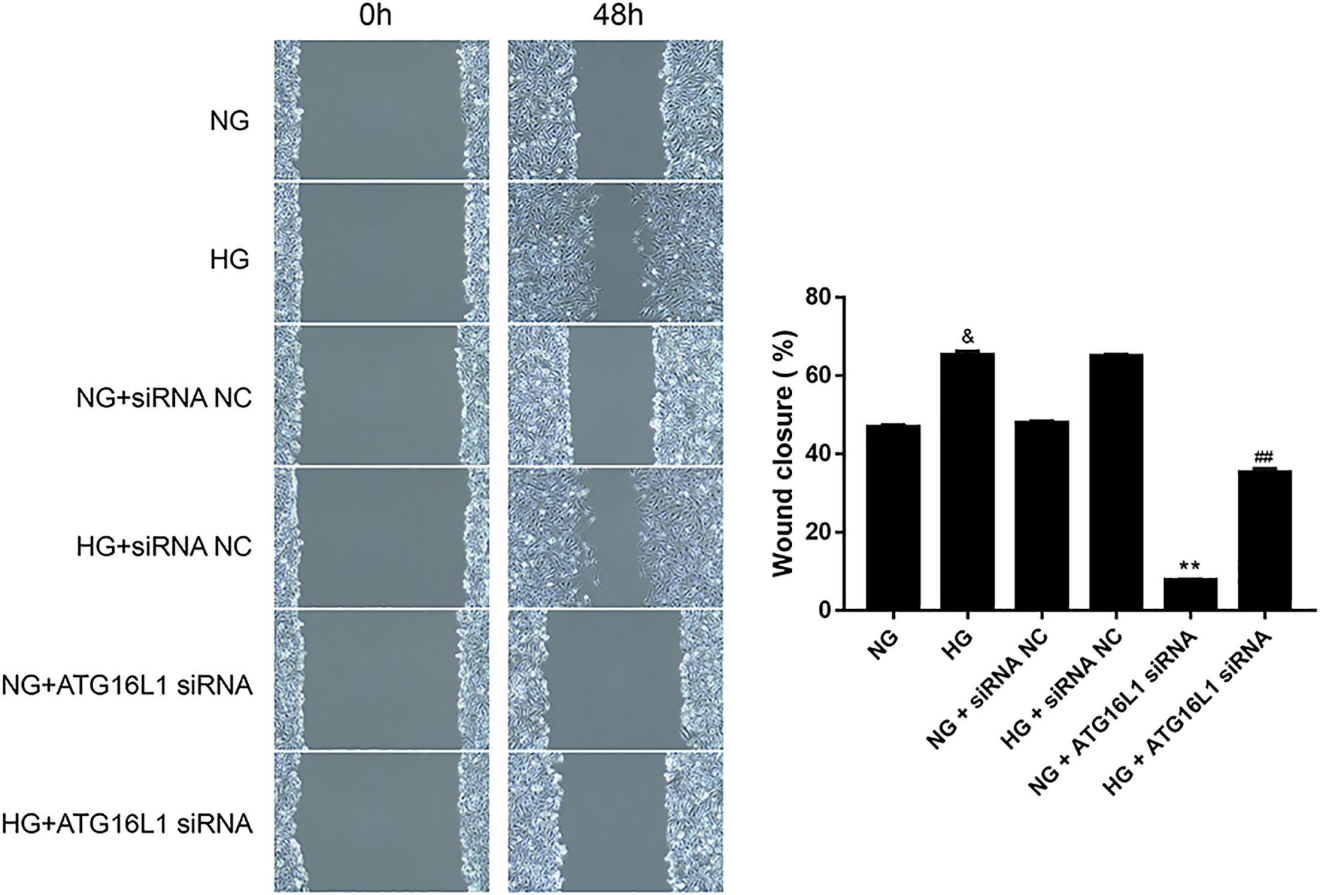
Compared with the control group, the migration ability of retinal capillary endothelial cells (RCECs) in both normal glucose (NG) and high glucose (HG) groups was remarkably inhibited after transfection with ATG16L1 siRNA (*n* = 5). &: *p* < 0.05, compared with the NG group. **: *p* < 0.01, compared with the NG + siRNA negative control (NC) group. ##: *p* < 0.01, compared with the HG + siRNA NC group.

We evaluated the migration of RCECs transfected with ATG16L1 siRNA or control siRNA after exposure to NG or HG for 48 h. By examining the wound area, we found that the migration ability of RCECs increased after HG treatment compared with NG treatment (Figure 3). Moreover, the migration ability of RCECs in the NG and HG groups decreased considerably after transfection with ATG16L1 siRNA compared with that in the control group (*p* < 0.01). This finding indicated that ATG16L1 plays a crucial role in promoting the migration of RCECs.

### Effect of ATG16L1 on the invasion ability of RCECs

**Figure 4:**
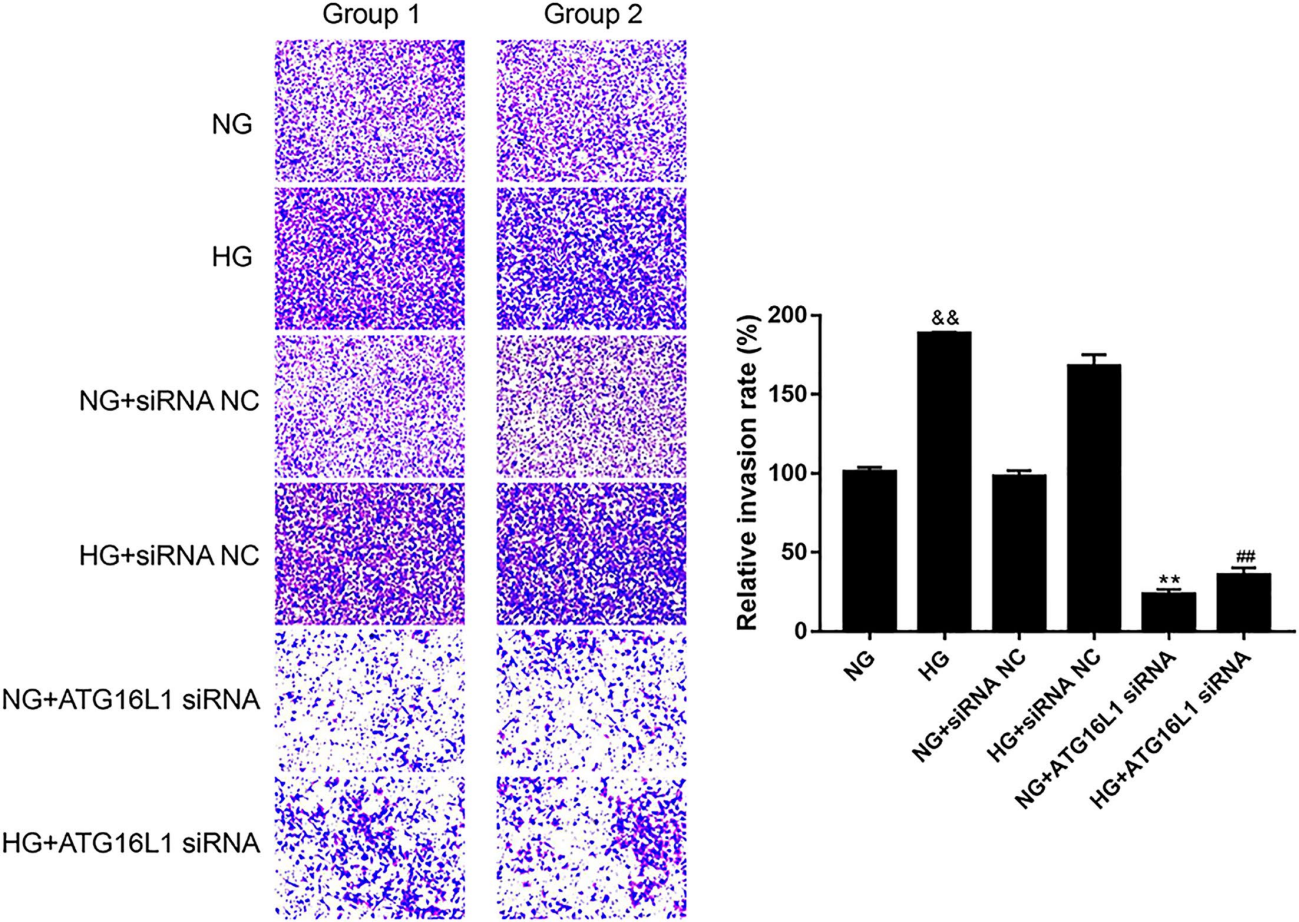
Compared with the control group, the invasion rate of retinal capillary endothelial cells (RCECs) in both high glucose (HG) and normal glucose (NG) groups was substantially suppressed after transfection with ATG16L1 siRNA (*n* = 5). &&: *p* < 0.01, compared with the NG group. **: *p* < 0.01, compared with the NG + siRNA negative control (NC) group. ##: *p* < 0.01, compared with the HG + siRNA NC group.

To further elucidate the role of ATG16L1 in cell invasion, we performed a Transwell migration assay. The results showed that the invasion rate of RCECs was significantly higher in the HG group than in the NG group (Figure 4). After transfection with ATG16L1 siRNA, the invasion rates of RCECs decreased significantly in the HG and NG groups compared with the control group (*p* < 0.01). This result indicated that ATG16L1 also enhanced the invasion ability of RCECs.

## Discussion

DR is a complication of diabetic neurodegeneration, inflammation, and microangiopathy. Several DR-related factors, such as nutritional stress, oxidative stress, hypoxia, and endoplasmic reticulum stress, are closely related to autophagy. Although our previous research demonstrated the potential involvement of ATG16L1 in the development and progression of DR (6), the expression and pathological role of ATG16L1 in DR remain largely unknown. In the present study, we conducted *in vitro* experiments to further investigate the mechanism of action of ATG16L1 in the pathogenesis of DR. The relative expression of ATG16L1 mRNA and protein was significantly upregulated in RCECs in the HG group compared with the NG group, whereas it was significantly downregulated in RCECs in the ATG16L1 siRNA group. Furthermore, the expression of ATG16L1 increased more significantly with the extension of time. In addition, the migration of RCECs was substantially suppressed in the NG and HG groups after transfection with ATG16L1 siRNA compared with that in the control group. These findings indicate that ATG16L1 influences the development of DR by promoting the migration of RCECs, thus further confirming our previous findings and hypothesis.

A previous study demonstrated that exposing the human immortalized RPE cell line ARPE-19 to HG levels might increase the number of autophagosomes. In cells cultured in HG medium, the number of autophagosomes increased, LC3-II levels increased, and P62 levels decreased compared with those in cells cultured in NG medium. HG-induced autophagy is primarily dependent on reactive oxygen species (ROS)–mediated regulation of endoplasmic reticulum stress (7). Another study used the same cell line cultured in HG medium and revealed a significant increase in autophagy flux and upregulation of LC3-II protein expression, indicating the effective activation of autophagy. Under the same experimental conditions, the inhibition of autophagy by 3-MA enhanced the activity of interleukin-1β and NLRP3, thereby reducing cell survival (8). Moreover, there was an increase in the expression levels of ATG12–ATG5 complex, ATG5, and ATG3 and the conversion of LC3B-I to LC3B-II under hyperglycemia in a retinal Müller cell line (9). Consistent with the abovementioned results, in the present study, the mRNA and protein expression levels of ATG16L1 increased significantly in cells exposed to HG compared with cells exposed to NG. These findings indicated that HG levels activated autophagy and that ATG16L1 expression was significantly upregulated in HG medium.

Some studies have investigated the role of autophagy in HG-induced neovascularization. For instance, Du et al. found that exposure to HG levels significantly reduced the viability of RF/6A cells and increased their migration and blood vessel formation. Additionally, 3-MA pretreatment increased cell viability, decreased angiogenesis and migration ability, and downregulated ROS accumulation and autophagy (10). However, the mechanism of action of ATG16L1 in DR has been rarely explored. Rajabinejad et al. conducted a comparative study using the peripheral blood of patients with and without diabetic optic neuropathy and revealed that ATG16L1 expression was significantly upregulated in patients with DN. Although DN symptoms are usually accompanied with DR symptoms, the abovementioned study showed that ATG16L1 in DN patients plays a vital role in visual acuity (11). The process of autophagy is divided into the following stages: initiation, elongation, maturation, and degradation. ATG16L1 participates in the elongation stage primarily through the formation of the ATG12–ATG5–ATG16L1 complex, which initially binds to LC-II and further binds to lysosomes to form autolysosomes, thus completing autophagy (12). mTOR is a negative regulatory factor of autophagy. In the early stage of DR, the reduction of retinal perfusion leading to tissue hypoxia may inhibit mTOR expression, thereby increasing autophagy and causing an increase in the release of vascular endothelial growth factor (VEGF) (12). Lopes de Faria et al. investigated the role of autophagy in regulating the response of retinal Müller cells (RMCs) to hyperglycemia and found that hyperglycemia promoted the initial step of RMC autophagy, which was indicated by the upregulation of beclin-1 and LC3-II protein levels. However, due to lysosomal dysfunction, the degradation process could not be completed, resulting in the accumulation of P62, which further led to the release of VEGF and apoptosis of RMCs (13). ATG16L1 is a key protein in the extension stage of autophagy. We believe that the regulatory effect of ATG16L1 on DR is similar to that reported by Lopes de Faria et al. because vascular endothelial cells are the main generating cells of VEGF, and ATG16L1 may affect the production of VEGF by regulating the apoptosis of endothelial cells (13). Ding et al. and Alshaman et al. also revealed an increase in the expression of LC-3II in DR (14, 15). Thus, we believe that ATG16L1 promotes the progression of autophagy by increasing the binding to LC-3II. Similarly, in our study, the mRNA and protein expression levels of ATG16L1 significantly decreased in the ATG16L1 siRNA group, along with a considerable decrease in the migration ability and invasion rate of RCECs. These findings indicate that ATG16L1 is involved in the pathogenesis of DR by enhancing the migration of cells. To summarize, we believe that the role of ATG16L1 in DR is to regulate the occurrence and development of DR simultaneously through two pathways—increasing autophagy in cells and increasing intracellular ROS production—thereby enhancing the release of VEGF. Our study mainly focused on primary rat RCECs, which are more representative than cell lines in the study of autophagy, and primarily conducted *in vitro* experiments related to the autophagy of RCECs. We intend to further conduct *in vivo* experiments to investigate the mechanism of action of ATG16L1 in DR in vivo. We also intend to further explore the specific mechanism of action and the downstream pathways regulated by ATG16L1 in an in vitro DR model. Further studies are warranted to confirm our findings in clinical settings.

## Conclusion

ATG16L1 may participate in the regulation of RCEC migration under HG conditions, indicating its potential role in the development of DR. Our study findings may help identify a novel therapeutic target.

## Data Availability

The raw data supporting the conclusions of this article will be made available by the authors, without undue reservation.
