# Supplementary figures and images for: ATG16L1 promotes cell migration and invasion in high glucose–induced retinal capillary endothelial cells
Source: Front Med (Lausanne). 2025 Jul 15;12:1515936. doi: 10.3389/fmed.2025.1515936 (PMC12303877; doi:10.3389/fmed.2025.1515936)

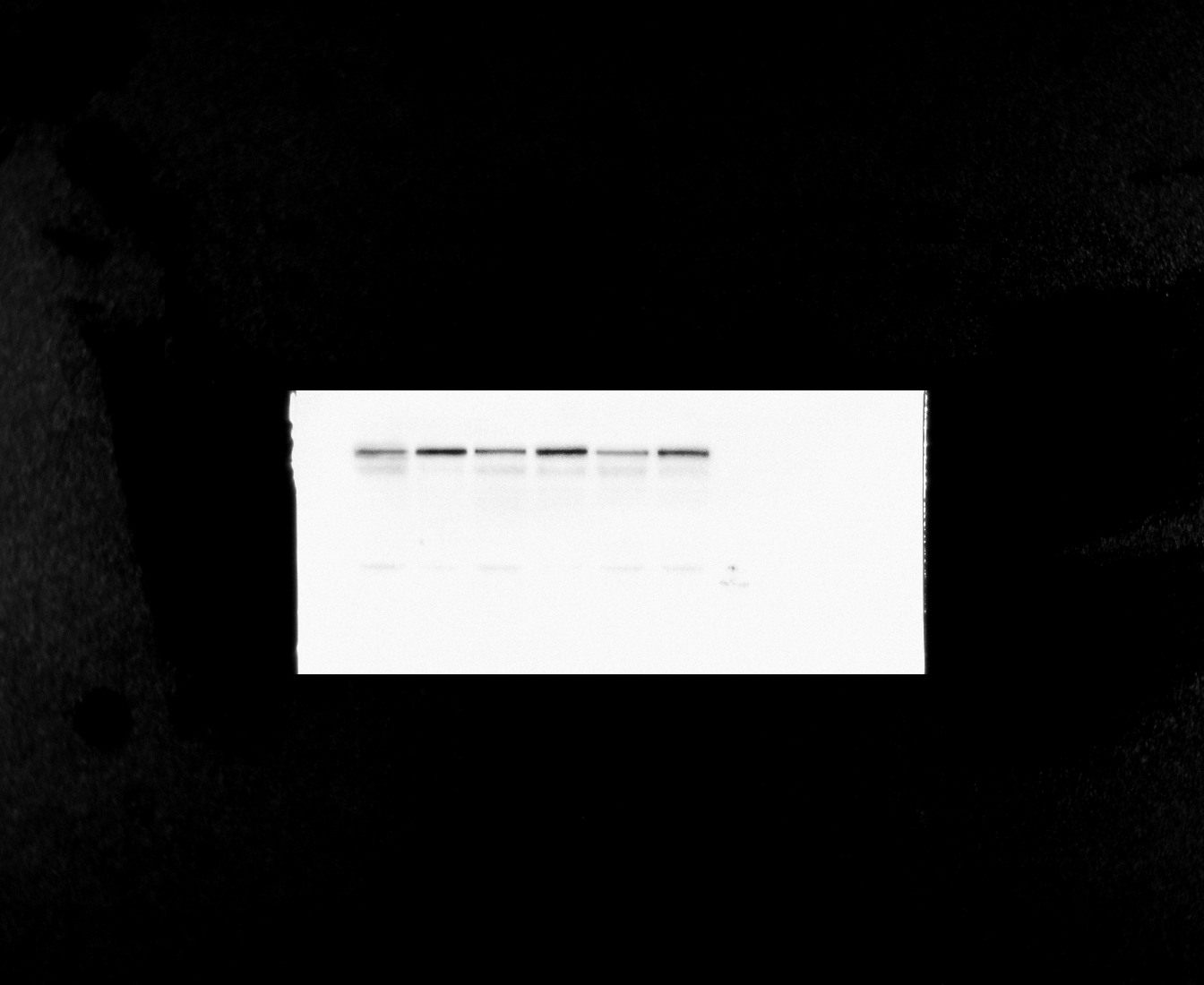

Supplement: Supplementary file 1 [file Image_1.TIF]

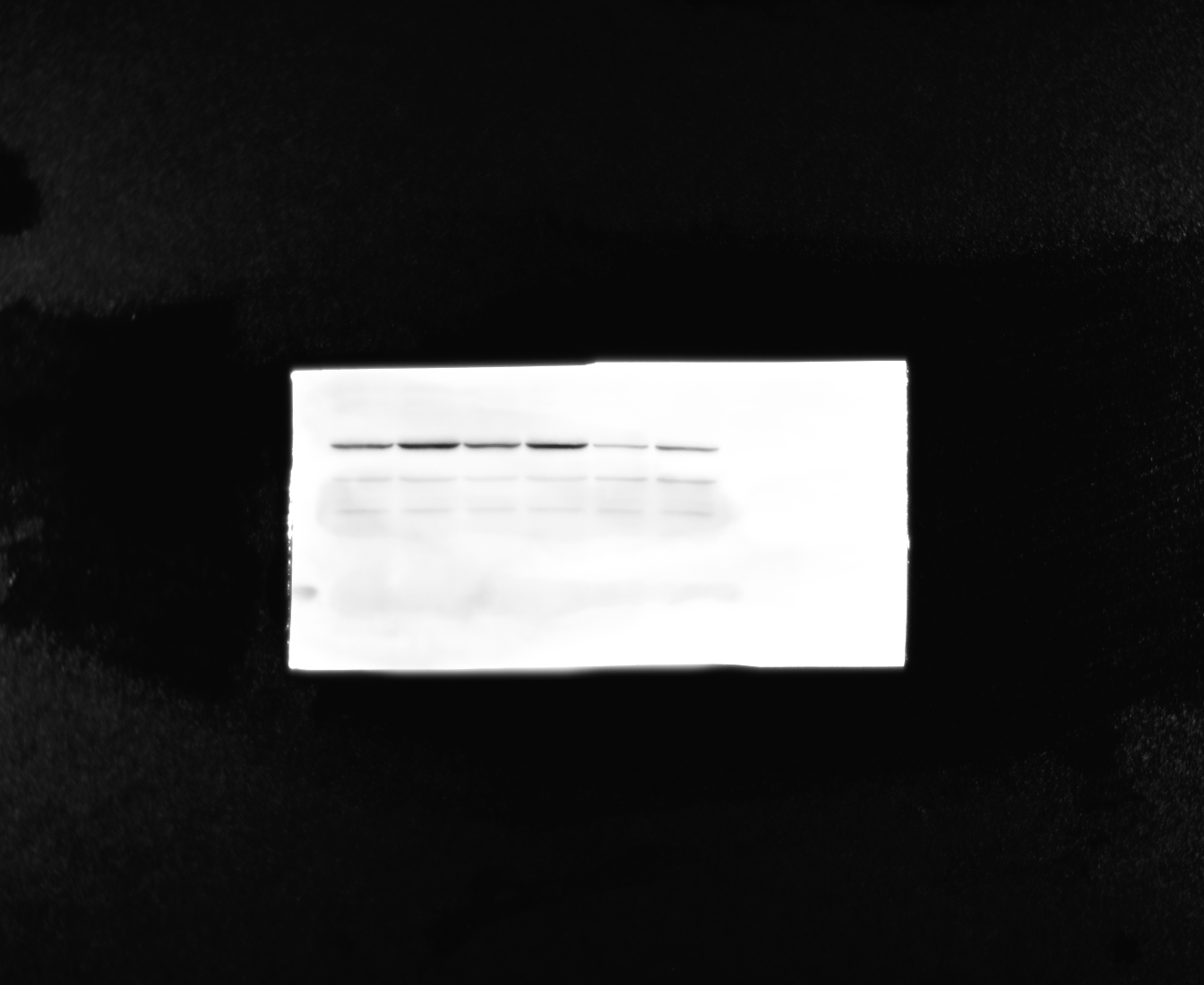

Supplement: Supplementary file 2 [file Image_2.TIF]
